# Programmed Death Ligand 1 (PDL1) Expression in Neoadjuvant Triple-Negative Breast Cancer: Association With Chemotherapy Response and Residual Cancer Burden

**DOI:** 10.1155/tbj/8856567

**Published:** 2025-09-30

**Authors:** Atif Ali Hashmi, Noreen Wahid, Ghazala Mudassir, Muhammad Irfan, Umair Arshad Malik, Erum Yousuf Khan, Syed Muhammad Abu Bakar, Naveen Faridi

**Affiliations:** ^1^Department of Histopathology, Liaquat National Hospital and Medical College, Karachi, Pakistan; ^2^Department of Pathology, Memorial Sloan Kettering Cancer Center, New York, New York, USA; ^3^Department of Pathology, Shifa College of Dentistry, Shifa Tameer-e-Millat University, Rawalpindi, Pakistan; ^4^Department of Statistics, Liaquat National Hospital and Medical College, Karachi, Pakistan; ^5^College of Osteopathic Medicine, Garden City Hospital, Michigan State University, Garden City, Michigan, USA

**Keywords:** chemotherapy response, predictive markers, prognostic markers, programmed death ligand 1 (PDL1), triple-negative breast cancer

## Abstract

**Background:**

Programmed death ligand 1 (PDL1) expression in tumors is linked to immune evasion in various cancers, making these patients potential candidates for PDL1 inhibitors. Although immune checkpoint blockade therapy has gained approval for breast cancer treatment, especially triple-negative breast cancer (TNBC), there is a lack of PDL1 expression data in Pakistani breast cancer patients. In our study, PDL1 expression was assessed in TNBC to determine eligibility for PDL1 inhibitors. Our study aimed to evaluate the frequency of PDL1 expression in TNBC. We also examined how PDL1 expression correlates with clinicopathological characteristics and prognostic factors in patients with TNBC. Moreover, the association of neoadjuvant chemotherapy response with PDL1 expression was also evaluated.

**Methods:**

This cross-sectional study was conducted at the Liaquat National Hospital Histopathology Department from January 2022 to June 2023. A total of 128 biopsy-proven cases of TNBCs were administered neoadjuvant chemotherapy before surgery during this period. PDL1 immunohistochemical staining was performed on prechemotherapy needle biopsies. Expression was determined using the combined positive score (CPS). CPS is the number of PDL1-stained cells (tumor cells, lymphocytes, and macrophages) divided by the total number of viable tumor cells multiplied by 100. Cases with CPS ≥ 10 were considered PDL1-positive.

**Results:**

Complete pathological response (pCR) was observed in 32.8% (*n* = 42) of cases. PDL1 expression was observed in 18.8% (*n* = 24) of cases. The majority of cases showed a high residual cancer burden (RCB-III) (*n* = 53, 41.4%). A significant association was noted between PDL1 expression and neoadjuvant chemotherapy response (*p* < 0.01). PDL1-positive cases had a higher pCR (*n* = 16, 66.7%) than PDL1-negative cases (*n* = 26, 25%). PDL1-positive cases showed a lower frequency of RCB-II-III (RCB-II: 8.3%; RCB-III: 0%) than PDL1-negative cases (RCB-II: 25%; RCB-III: 51%), with a significant *p* value (*p* < 0.01).

**Conclusion:**

Overall, PDL1 expression was low in TNBC cases in our study; however, identifying these cases is important to identify those that can benefit from immunotherapy. We found a significant association of PDL1 expression with neoadjuvant chemotherapy response and RCB. Moreover, PDL1 positivity was associated with lower Ki67 index and older age. Therefore, we recommend routine PDL1 testing in all cases of TNBC to predict neoadjuvant chemotherapy response.

## 1. Introduction

Breast cancer (BC) remains a significant concern for women's health and overall well-being. Because of early detection and treatment, there has been a marked (42%) decline in the mortality rate associated with BC through the year 2021 [1]. Despite recent progress, approximately 20% of individuals diagnosed with BC continue to experience the development of metastases, ultimately leading to fatal disease progression [2]. Over the past few decades, scientists have pinpointed molecular alterations linked to mammary oncogenesis and metastatic advancement, resulting in noteworthy therapeutic breakthroughs. These advancements encompass hormone therapy designed to target the estrogen receptor (ER) and specialized treatments aimed at oncogenic proteins. However, in most cases, resistant clones emerge if they are not initially present, owing to the highly mutagenic and adaptable nature of cancer cells. This adaptability renders tumor responses only temporary. Theoretically, cancer immunotherapy can address this issue because of the adaptability of the immune response. Despite the conventional view that BC is not inherently “immunogenic,” there is a significant association between favorable outcomes and the presence of tumor-infiltrating lymphocytes (TILs) in various studies. More specifically, the existence of lymphocytes in the tumor microenvironment and gene expression profiles representing these cells consistently correlate with a slightly but significantly improved prognosis. This correlation is particularly pronounced in high-grade and ER-negative tumors [3–5]. New evidence emphasizes the vital function of the programmed cell death receptor pathway in maintaining an immunosuppressive environment within tumors [6]. Programmed cell death protein 1 (PD-1) is found in various immune cell types, and its activation hampers the function, survival, and expansion of T cells. The PD-1 ligand, programmed death ligand 1 (PDL1), is present on activated T cells, B cells, dendritic cells, and macrophages, as well as in certain immune-privileged nonhematopoietic tissues [7, 8]. PDL1 expression can occur in tumors from different locations, including those originating in the breast, ovaries, stomach, pancreas, lungs, and renal cells [9–12]. The inhibition of local immune responses by tumor cells through PDL1 expression is believed to shield the tumor from T cell-mediated killing. Clinical trials in the early phases employing monoclonal antibodies targeting PD-1 or PDL1 have revealed substantial and enduring clinical responses in patients with resistant solid tumors [13, 14].

The advent of immunotherapy and antibody–drug conjugates (ADCs) has changed the landscape of BC, especially triple-negative BC (TNBC), for which conventional treatment options are limited. However, response to these management strategies is governed by several predictors, such as PDL1 status, TILs, and tumor mutational burden, especially in Stage I–III TNBC [15]. Similarly, the role of ADCs such as, datopotecan–deruxtecan has also shown promising results in TNBC, which further underscores the need to evaluate predictive markers in this setting [16]. In addition to the role in primary settings, the effectiveness of these novel therapies, such as sacituzumab govitecan has also been established in metastatic TNBC [17]. In this context, the significance of cachexia index and the link between cancer and inflammation/metabolic syndromes should also be considered [18, 19].

Studies on the PD1–PDL1 pathway in BC are limited. Only a few studies have been conducted to correlate prognostic significance and predictive value in TNBC; however, the results vary to a great degree [5, 20, 21]. Therefore, in this study, we sought the frequency of expression of PDL1 in TNBC in our population and its association with chemotherapy response and other prognostic parameters.

## 2. Materials and Methods

### 2.1. Patients and Methods

This cross-sectional study was conducted at the Histopathology Department of the Liaquat National Hospital from January 2022 to October 2023. The Ethical Review Committee of the Liaquat National Hospital approved the study. A total of 128 biopsy-proven cases of TNBC that were administered neoadjuvant chemotherapy before surgery were included in the study. The histopathological type and grade were determined on prechemotherapy needle biopsy specimens. Similarly, ER, progesterone receptor (PR), human epidermal growth factor receptor 2 (HER2neu), and Ki67 immunohistochemical (IHC) expression were determined on prechemotherapy biopsy specimens. ER and PR were considered negative if less than 1% nuclear expression was noted. Complete strong membranous expression of > 10% invasive cancer cells was considered positive for HER2neu expression. Equivocal HER2neu IHC staining was confirmed by fluorescence in situ hybridization (FISH). Cases with negative ER, PR, and HER2neu (0 or 1+ IHC or 2+ IHC and FISH nonamplified) were included in the study.

### 2.2. PDL1 IHC Expression

**Figure 1:**
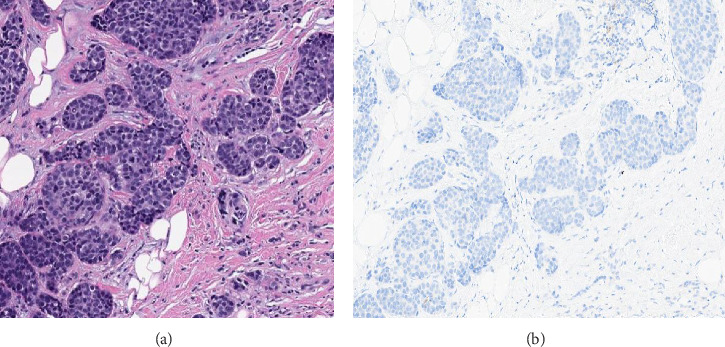
(a) Hematoxylin and eosin–stained section with invasive ductal carcinoma, Grade II. (b) Negative PDL1 expression (CPS score = 0).

**Figure 2:**
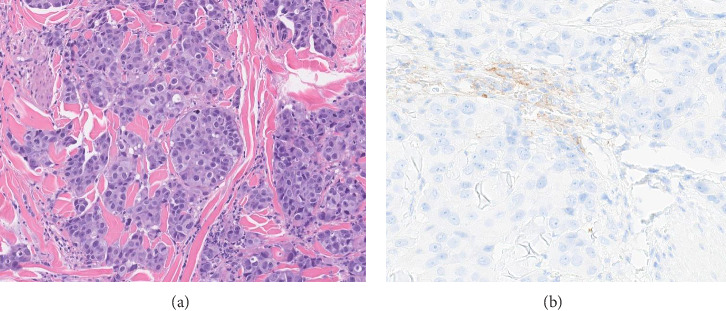
(a) Hematoxylin and eosin–stained section with invasive ductal carcinoma, Grade III. (b) Negative PDL1 expression (CPS score = 2).

**Figure 3:**
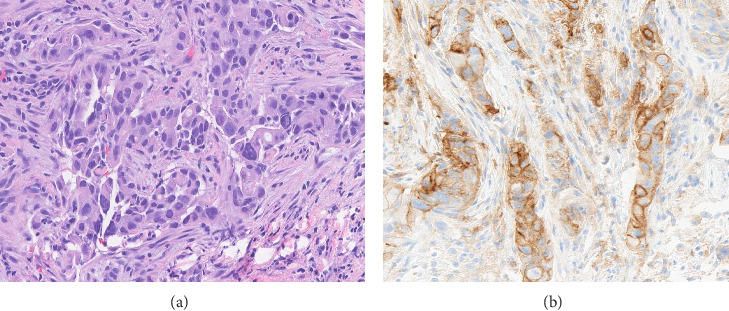
(a) Hematoxylin and eosin–stained section with invasive ductal carcinoma, Grade III. (b) Positive PDL1 expression (CPS score = 30).

**Figure 4:**
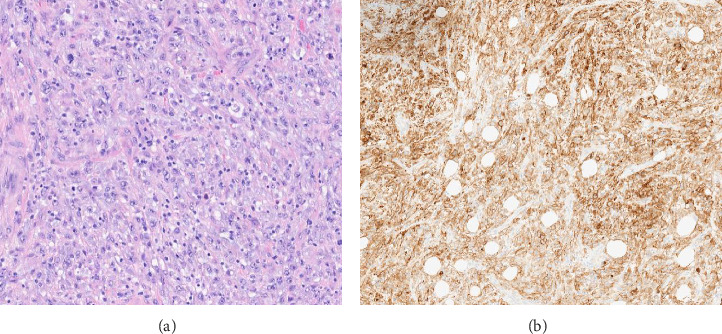
(a) Hematoxylin and eosin–stained section with metaplastic carcinoma (spindle cell), Grade III. (b) Positive PDL1 expression (CPS score > 90).

PDL1 IHC staining was performed on prechemotherapy needle biopsies. Expression is determined using the combined positive score (CPS). CPS is the number of PDL1-stained cells (tumor cells, lymphocytes, AND macrophages) divided by the total number of viable tumor cells, multiplied by 100. Specimens were considered to have positive PDL1 expression if CPS is ≥ 10 [22]. Cases with CPS < 10 were considered negative for PDL1 expression (Figures 1, 2, 3, and 4).

### 2.3. Neoadjuvant Chemotherapy Response in TNBC

After completion of neoadjuvant chemotherapy, definite surgical resection was performed. The following neoadjuvant therapy regime was followed in all cases: Preoperative pembrolizumab 3 times weekly (4 cycles) plus carboplatin or paclitaxel for 12 weeks. This was followed by pembrolizumab along with Adriamycin and cyclophosphamide 3 times weekly (4 cycles), followed by surgical resection. Resection specimens (lumpectomy, mastectomy with sentinel lymph node dissection or modified radical mastectomy) were sent to the histopathology laboratory. All surgical resection specimens had a negative resection margin for tumor (R0 resection). Cases with positive resection margin underwent additional resection to obtain a negative clearance margin (for lumpectomy and breast conservation surgeries).

Grossing of the specimens was performed in the histopathology laboratory. Representative sections were obtained from the tumor bed, and the pathological response to chemotherapy was assessed. Neoadjuvant chemotherapy response was categorized as pCR, when no residual invasive BC (IBC) was found on postchemotherapy excision specimen in either breast or lymph nodes, and pathological partial response (pPR), when residual cancer cells were present with admixed chemotherapy-related changes. Residual cancer burden (RCB) was evaluated using the MD Anderson calculator for assessing RCB (https://www.mdanderson.org/breastCancer_RCB). Cases with pCR were assigned RCB-0; cases with RCB ranging from > 0 to ≤ 1.36 were labeled as RCB-I. Cases with RCB between 1.37 and 3.28 were called RCB-II, and cases with RCB > 3.28 were assigned RCB-III [23]. Association of chemotherapy response with PDL1 expression was evaluated.

### 2.4. Statistical Analysis

Data analysis was performed using the Statistical Package for Social Sciences (Version 26.0, IBM Corp., Armonk, NY, USA). The chi-square test, independent *t*-test, and Fisher's exact test were used to seek the association between categorical variables. *p* values < 0.05 were considered significant.

## 3. Results

### 3.1. Clinicopathological Parameters of the Study Population

Table 1 shows the clinicopathological parameters of the study population. Most tumors were IBC, no special type (IBC-NST), accounting for 90.6% (*n* = 116) of cases. Most patients were < 50 years old (78.1.1%). The mean Ki67 index was high (44.32 ± 21.48). pCR was observed in 32.8% (*n* = 42) of cases. PDL1 expression was observed in 18.8% (*n* = 24) of cases. The majority of cases showed a high RCB-III (*n* = 53, 41.4%).

### 3.2. Association of PDL1 Expression With Clinicopathological Parameters

Table 2 shows the association between PDL1 expression and the clinicopathological parameters. Notably, a significant association was found between PDL1 expression and age, Ki67 index, and RCB. PDL1 positivity was associated with older age and a lower Ki67 index. PDL1-positive cases showed a significantly lower RCB (mean ± SD: 0.47 ± 0.75) than PDL1-negative cases (mean ± SD: 2.86 ± 1.88).

### 3.3. Association of PDL1 Expression With Neoadjuvant Chemotherapy Response

**Figure 5:**
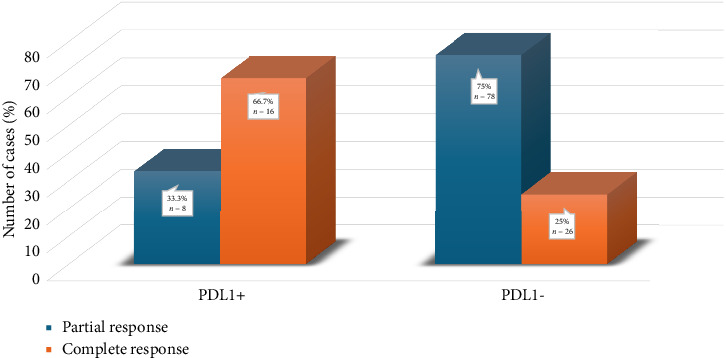
Association of PDL1 expression with neoadjuvant chemotherapy response.

Figure 5 shows the association of PDL1 expression with neoadjuvant chemotherapy response. A significant association was noted between PDL1 expression and neoadjuvant chemotherapy response (*p* < 0.01). PDL1-positive cases had a higher pCR (*n* = 16, 66.7%) than PDL1-negative cases (*n* = 26, 25%).

### 3.4. Association of PDL1 Expression With RCB

**Figure 6:**
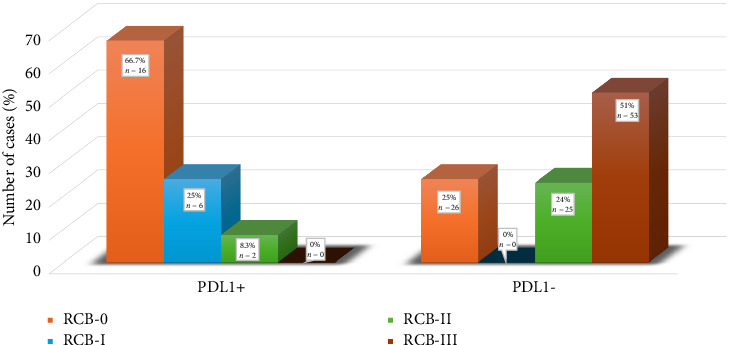
Association of PDL1 expression with residual cancer burden (RCB).

Figure 6 depicts the association of PDL1 expression with RCB. PDL1-positive cases showed a lower frequency of RCB-II-III (RCB-II: 8.3%; RCB-III: 0%) than PDL1-negative cases (RCB-II: 25%; RCB-III: 51%), with a significant *p* value (*p* < 0.01).

## 4. Discussion

We found a low expression of PDL1 in our cohort of TNBC cases. We found a significant association of PDL1 expression with RCB after neoadjuvant chemotherapy and pCR. PDL1-positive cases had a lower RCB score and a higher frequency of pCR than PDL1-negative cases, highlighting the predictive value of PDL1 in assessing response to neoadjuvant chemotherapy.

Pakistan has the highest BC incidence rate in Asia, with one in nine women at risk of being diagnosed during their lifetime, and young women often present at advanced stages, adversely impacting prognosis [24, 25]. Studying BC patients from Pakistan, who differ demographically from Western populations, is important. PDL1, the key ligand of PD-1, is naturally expressed in myeloid, lymphoid, and normal epithelial cells [26]. PDL1 expression has been observed in BC but not in normal breast tissue [27]. In numerous clinical studies, there is growing evidence supporting the connection between PDL1 tumor expression and adverse prognostic features as well as high-risk clinicopathological parameters in BC patients. In our study, a positive correlation was observed between PDL1 expression on tumor cells and advancing age and a lower Ki67 index. Multiple prior studies have indicated associations between PDL1 expression and adverse clinicopathological characteristics, such as higher grade, increased tumor size, lymph node metastasis, lymphovascular invasion (LVI), and hormone receptor negativity [6, 28–31]. Qin et al. studied 870 BC patients and showed that PDL1 positivity was associated with TNBC and overall survival [31]. However, another study conducted by Kim et al. showed that intratumoral PDL1 expression was associated with favorable outcomes in patients with TNBC and showed better disease-free survival (DFS) [32].

With the discovery and approval of more and more immunotherapies, there is an inclement to find histological and IHC markers to predict response to immunotherapy. A recent review by Angelico et al. discussed the role of TILs histologically and PDL1 IHC in TNBC that can predict response to immunotherapy [33]. Overall, PDL1 positivity is more in TNBC and HER2neu-positive BCs, while it is low in hormone-positive BCs. A major hindrance and reason behind this variability is the lack of an established cut-off to define PDL1 positivity [32]. We found a low positivity of PDL1 in our cohort of cases of TNBC, as we took a CPS score of 10 or above to define a positive PDL1 expression. It is crucial to accurately define a cut-off PDL1 CPS score as it is becoming one of the defining criteria for immunotherapy eligibility in metastatic TNBC.

In the current study, we comprehensively evaluated the predictive role of PDL1 in neoadjuvant TNBC. Based on the study results, the upfront evaluation of this biomarker is important to guide management strategy in TNBC. However, guidelines regarding the use of PDL1 in the primary setting are not established. Similarly, the predictive value of PDL1 with newer ADCs is lacking, and the association of PDL1 with the tumor microenvironment and molecular profile is also not delineated. Future research on this topic should focus on the association of PDL1 expression with other biomarkers, molecular BC profile, tumor mutational burden, evolution of PDL1 expression in recurrent and metastatic TNBC, and the predictive role of PDL1 with ADCs. The future of immunotherapy and ADCs largely depends on personalized biomarker and molecular profiles of TNBC, and this area is rapidly evolving to encompass numerous biomarkers, apart from PDL1.

### 4.1. Limitations

This study has some limitations. First, the data that were collected were retrospective and from a single institution. Second, long-term follow-up was lacking to delineate cases that recur over the period. Furthermore, the predictive response of PDL1 in metastatic settings and ADCs was not evaluated in our study. In addition, the predictive response of TILs and their association with PDL1 expression were not assessed in this study.

## 5. Conclusion

Overall, PDL1 expression was low in TNBC cases in our study; however, identifying these cases is important to identify those that can benefit from immunotherapy. We found a significant association of PDL1 expression with neoadjuvant chemotherapy response and RCB. Moreover, PDL1 positivity was associated with lower Ki67 index and older age. Therefore, we recommend routine PDL1 testing in all cases of TNBC to predict neoadjuvant chemotherapy response.

## Figures and Tables

**Table 1:** Clinicopathological parameters of the population under study.

Clinicopathological parameters	Values
Age (years); mean ± SD	46.90 ± 10.99
Age group	
≤ 50 years, *n* (%)	100 (78.1)
> 50 years, *n* (%)	28 (21.9)
Ki67 (%); mean ± SD	44.32 ± 21.48
Ki67 group	
≤ 15%, *n* (%)	15 (11.7)
16%–30%, *n* (%)	26 (20.3)
> 30%, *n* (%)	87 (68)
Histologic type	
Invasive breast carcinoma NST/ductal carcinoma, *n* (%)	116 (90.6)
Others, *n* (%)	12 (9.4)
Nodal metastasis	
Present, *n* (%)	74 (57.8)
Absent, *n* (%)	54 (42.2)
Procedure	
Breast conservation surgery, *n* (%)	12 (9.4)
Lumpectomy, *n* (%)	32 (25)
Mastectomy with sentinel lymph node dissection, *n* (%)	6 (4.7)
Modified radical mastectomy, *n* (%)	50 (39.1)
Simple mastectomy, *n* (%)	28 (21.9)
Laterality	
Right, *n* (%)	80 (62.5)
Left, *n* (%)	48 (37.5)
Pathological response to neoadjuvant therapy	
Pathological partial response, *n* (%)	86 (67.2)
Pathological complete response, *n* (%)	42 (32.8)
Tumor grade	
Grade 1–2, *n* (%)	56 (43.8)
Grade 3, *n* (%)	72 (56.3)
RCB score; mean ± SD	2.41 ± 1.96
RCB classification	
RCB-0	42 (32.8)
RCB-I	6 (4.7)
RCB-II	27 (21.1)
RCB-III	53 (41.4)
PDL1 expression	
Positive, *n* (%)	24 (18.8)
Negative, *n* (%)	104 (81.3)

Abbreviations: NST, no special type; PDL1, programmed death ligand 1; SD, standard deviation.

**Table 2:** Association of PDL1 expression with clinicopathological parameters.

Clinicopathological parameters	Values		*p* value
	PDL1 expression		
	Positive	Negative	
Age (years); mean ± SD^∗^	55.25 ± 9.16	44.98 ± 10.50	< 0.001^∗∗∗^
Age group^∗∗^			
≤ 50 years, *n* (%)	13 (54.2)	87 (83.7)	0.002^∗^
> 50 years, *n* (%)	11 (45.8)	17 (16.3)	
Ki67 (%); mean ± SD^∗^	30.83 ± 10.17	47.43 ± 22.22	< 0.001^∗∗∗^
Ki67 group^∗∗^			
≤ 15%, *n* (%)	0 (0)	15 (14.4)	0.001^∗^
16%–30%, *n* (%)	11 (45.8)	15 (14.4)	
> 30%, *n* (%)	13 (54.2)	74 (71.2)	
Histological type^∗∗^			
Invasive ductal carcinoma, *n* (%)	24 (100)	92 (88.5)	0.121
Others, *n* (%)	0 (0)	12 (11.5)	
Nodal metastasis^∗∗^			
Present, *n* (%)	11 (45.8)	63 (60.6)	0.187
Absent, *n* (%)	13 (54.2)	41 (39.4)	
Tumor grade^∗∗^			
Grade 1–2, *n* (%)	11 (45.8)	45 (43.3)	0.819
Grade 3, *n* (%)	13 (54.2)	59 (56.7)	
RCB score; mean ± SD^∗^	0.47 ± 0.75	2.86 ± 1.88	< 0.001^∗∗∗^

^∗^Independent *t*-test was applied.

^∗∗^Chi-square/Fisher's exact test was applied.

^∗∗∗^Significant at 0.05 level.

## Data Availability

The data are available from the corresponding author upon request.

## References

[1] https://www.cancer.org/cancer/types/breast-cancer/about/how-common-is-breast-cancer.html.

[2] Emens L. A. (2018). Breast Cancer Immunotherapy: Facts and Hopes. *Clinical Cancer Research*.

[3] Denkert C., Loibl S., Noske A. (2010). Tumor-Associated Lymphocytes as an Independent Predictor of Response to Neoadjuvant Chemotherapy in Breast Cancer. *Journal of Clinical Oncology*.

[4] Loi S., Michiels S., Salgado R. (2014). Tumor Infiltrating Lymphocytes are Prognostic in Triple Negative Breast Cancer and Predictive for Trastuzumab Benefit in Early Breast Cancer: Results From the FinHER Trial. *Annals of Oncology*.

[5] Schalper K. A., Velcheti V., Carvajal D. (2014). In Situ Tumor PD-L1 mRNA Expression is Associated With Increased TILs and Better Outcome in Breast Carcinomas. *Clinical Cancer Research*.

[6] Sabatier R., Finetti P., Mamessier E. (2015). Prognostic and Predictive Value of PDL1 Expression in Breast Cancer. *Oncotarget*.

[7] Schreiber R. D., Old L. J., Smyth M. J. (2011). Cancer Immunoediting: Integrating Immunity’s Roles in Cancer Suppression and Promotion. *Science*.

[8] Nurieva R. I., Liu X., Dong C. (2009). Yin-Yang of Costimulation: Crucial Controls of Immune Tolerance and Function. *Immunological Reviews*.

[9] Ghebeh H., Mohammed S., Al-Omair A. (2006). The B7-H1 (PD-L1) T Lymphocyte-Inhibitory Molecule is Expressed in Breast Cancer Patients With Infiltrating Ductal Carcinoma: Correlation With Important High-Risk Prognostic Factors. *Neoplasia*.

[10] Hamanishi J., Mandai M., Iwasaki M. (2007). Programmed Cell Death 1 Ligand 1 and Tumor-Infiltrating CD8+ T Lymphocytes are Prognostic Factors of Human Ovarian Cancer. *Proceedings of the National Academy of Sciences of the USA*.

[11] Wu C., Zhu Y., Jiang J., Zhao J., Zhang X. G., Xu N. (2006). Immunohistochemical Localization of Programmed Death-1 Ligand-1 (PD-L1) in Gastric Carcinoma and Its Clinical Significance. *Acta Histochemica*.

[12] Nomi T., Sho M., Akahori T. (2007). Clinical Significance and Therapeutic Potential of the Programmed Death-1 Ligand/Programmed Death-1 Pathway in Human Pancreatic Cancer. *Clinical Cancer Research*.

[13] Brahmer J. R., Tykodi S. S., Chow L. Q. (2012). Safety and Activity of Anti-PD-L1 Antibody in Patients With Advanced Cancer. *New England Journal of Medicine*.

[14] Topalian S. L., Hodi F. S., Brahmer J. R. (2012). Safety, Activity, and Immune Correlates of Anti-PD-1 Antibody in Cancer. *New England Journal of Medicine*.

[15] Rizzo A., Cusmai A., Acquafredda S. (2022). KEYNOTE-522, IMpassion031 and GeparNUEVO: Changing the Paradigm of Neoadjuvant Immune Checkpoint Inhibitors in Early Triple-Negative Breast Cancer. *Future Oncology*.

[16] Schipilliti F. M., Drittone D., Mazzuca F., La Forgia D., Guven D. C., Rizzo A. (2024). Datopotamab Deruxtecan: A Novel Antibody Drug Conjugate for Triple-Negative Breast Cancer. *Heliyon*.

[17] Caputo R., Buono G., Piezzo M. (2024). Sacituzumab Govitecan for the Treatment of Advanced Triple Negative Breast Cancer Patients: A Multi-Center Real-World Analysis. *Frontiers in Oncology*.

[18] Bas O., Sahin T. K., Karahan L., Rizzo A., Guven D. C. (2025). Prognostic Significance of the Cachexia Index (CXI) in Patients With Cancer: A Systematic Review and Meta-Analysis. *Clinical Nutrition ESPEN*.

[19] Vitale E., Rizzo A., Santa K., Jirillo E. (2024). Associations Between “Cancer Risk”, “Inflammation” and “Metabolic Syndrome”: A Scoping Review. *Biology*.

[20] Loi S., Sirtaine N., Piette F. (2013). Prognostic and Predictive Value of Tumor-Infiltrating Lymphocytes in a Phase III Randomized Adjuvant Breast Cancer Trial in Node-Positive Breast Cancer Comparing the Addition of Docetaxel to Doxorubicin With Doxorubicin-Based Chemotherapy: BIG 02-98. *Journal of Clinical Oncology*.

[21] Mittendorf E. A., Philips A. V., Meric-Bernstam F. (2014). PD-L1 Expression in Triple-Negative Breast Cancer. *Cancer Immunology Research*.

[22] https://www.pathologyoutlines.com/topic/stainsPDL1.html.

[23] Yau C., Osdoit M., van der Noordaa M. (2022). Residual Cancer Burden After Neoadjuvant Chemotherapy and Long-Term Survival Outcomes in Breast Cancer: A Multicentre Pooled Analysis of 5161 Patients. *The Lancet Oncology*.

[24] Menhas R., Umer S. (2015). Breast Cancer Among Pakistani Women. *Iranian Journal of Public Health*.

[25] Khan N. H., Duan S. F., Wu D. D., Ji X. Y. (2021). Better Reporting and Awareness Campaigns Needed for Breast Cancer in Pakistani Women. *Cancer Management and Research*.

[26] Kythreotou A., Siddique A., Mauri F. A., Bower M., Pinato D. J. (2018). PD-L1. *Journal of Clinical Pathology*.

[27] Li S., Chen L., Jiang J. (2019). Role of Programmed Cell Death Ligand-1 Expression on Prognostic and Overall Survival of Breast Cancer: A Systematic Review and Meta-Analysis. *Medicine (Baltimore)*.

[28] Huang W., Ran R., Shao B., Li H. (2019). Prognostic and Clinicopathological Value of PD-L1 Expression in Primary Breast Cancer: A Meta-Analysis. *Breast Cancer Research and Treatment*.

[29] Dill E. A., Gru A. A., Atkins K. A. (2017). PD-L1 Expression and Intratumoral Heterogeneity Across Breast Cancer Subtypes and Stages: An Assessment of 245 Primary and 40 Metastatic Tumors. *The American Journal of Surgical Pathology*.

[30] Tawfik O., Kimler B. F., Karnik T., Shehata P. (2018). Clinicopathological Correlation of PD-L1 Expression in Primary and Metastatic Breast Cancer and Infiltrating Immune Cells. *Human Pathology*.

[31] Qin T., Zeng Y. D., Qin G. (2015). High PD-L1 Expression was Associated With Poor Prognosis in 870 Chinese Patients With Breast Cancer. *Oncotarget*.

[32] Hyun-Soo K. I. M., Sung-Im D. O., Dong-Hoon K. I. M., Sophia A. (2020). Clinicopathological and Prognostic Significance of Programmed Death Ligand 1 Expression in Korean Patients With Triple-Negative Breast Carcinoma. *Anticancer Research*.

[33] Angelico G., Broggi G., Tinnirello G. (2023). Tumor Infiltrating Lymphocytes (TILS) and PD-L1 Expression in Breast Cancer: A Review of Current Evidence and Prognostic Implications From Pathologist’s Perspective. *Cancers (Basel)*.

